# NEF-DHR: A Non-Equivalent Functional Dynamic Heterogeneous Redundancy Architecture for Endogenous Safety and Security

**DOI:** 10.3390/e28040463

**Published:** 2026-04-17

**Authors:** Bingbing Jiang, Yilin Kang, Hanzhi Cai

**Affiliations:** 1 Department of Computer Science and Technology, Huaiyin Normal University, Huai’an 223300, China; jiangbingbing@hytc.edu.cn; 2 School of Engineering, Yuxi Normal University, Yuxi 653100, China; 3 Yunnan Key Laboratory of Smart City in Cyberspace Security, Yuxi 653100, China; 4 Purple Mountain Laboratories, Nanjing 211111, China; caihanzhi@pmlabs.com.cn

**Keywords:** function secret sharing, non-equivalent functional decomposition, information entropy, DHR architecture, endogenous safety and security

## Abstract

Endogenous safety and security (ESS), which advocates for designing systems that are inherently safe and secure by nature, has emerged as a pivotal paradigm for addressing the inherent vulnerabilities of information systems. The Dynamic Heterogeneous Redundancy (DHR) architecture serves as its typical implementation by introducing dynamic, heterogeneous, redundant executors with equivalent function (EF) into the information system. However, the functional equivalence property explicitly connects the system’s output to that of the individual executors, thereby creating potential security risks that adversaries could exploit. In addition, EF-DHR faces an inherent contradiction between functional equivalence and heterogeneous implementations (HIS), leading to high engineering costs and limited applicability. To address these problems, this paper proposes the Non-Equivalent Functional DHR (NEF-DHR) architecture, leveraging function secret sharing (FSS) theory to replace EF executors with NEF components, which fundamentally eliminates the EF-HIS contradiction. Specifically, we propose the concept of ‘terminal executor output information entropy loss’ to formalize the risk of output information interception by adversaries and theoretically prove that NEF-DHR improves unpredictability and resistance to attacks. Experimental results further validate that NEF-DHR exhibits lower error rates under various attack levels, with enhanced robustness and superior ESS performance. Additionally, we generalize the DHR architecture based on three core properties (indistinguishability, output recoverability, verification) and classify ESS into three types with corresponding DHR variants. This work advances the application of entropy theory in ESS and provides a novel entropy-enhanced solution for the large-scale deployment of DHR security systems.

## 1. Introduction

**Figure 1:**
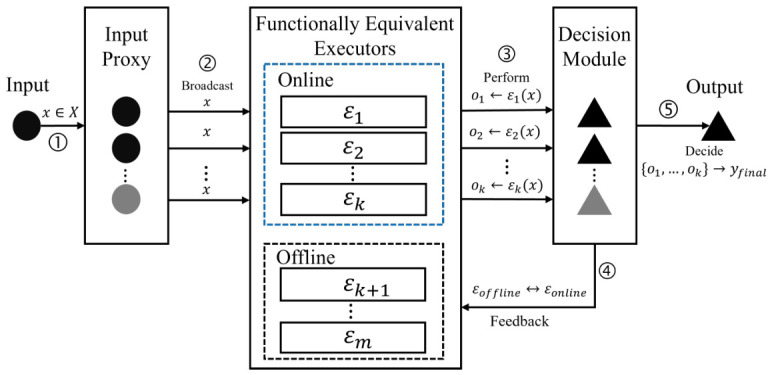
Traditional (equivalent functional) DHR (EF-DHR) architecture for endogenous safety and security. *Data flow: system input x*, intermediate output $o_{i}$, final output $y_{final}$, and online/offline executor switch; computational cost: O(1) input proxy, O(|f|) single executor, O(k) decision module for majority voting, and O(m) scheduling; security boundaries: inner security boundary encompasses all executors to protect heterogeneous implementation core, and outer security boundary encompasses input proxy and decision module to protect system input/output and dynamic scheduling; attack surfaces: input proxy (sniffing input *x*), executor communication link (sniffing $o_{i}$, EF leads to full information leakage per Remark 1), and decision module (manipulating voting strategy and tampering with executor switch logic).

The rapid proliferation of complex information systems in cyberspace has brought unprecedented convenience, but it has also exacerbated security and safety risks, characterized by inherent vulnerabilities and backdoors that are difficult to eradicate [1,2,3,4]. Traditional perimeter-based security measures (e.g., firewalls [5,6,7] and intrusion detection systems [8,9,10]) are ineffective in addressing these endogenous risks, as they merely suppress surface-level threats rather than addressing the root cause of vulnerability evolution. In this context, the endogenous safety and security (ESS) theory [11] has been proposed, redefining vulnerabilities and backdoors as intrinsic attributes of information systems and advocating for security guarantees derived from internal system dynamics rather than external additions. As a core implementation of ESS, the Dynamic Heterogeneous Redundancy (DHR) architecture leverages the principle of ‘functional equivalence and heterogeneous implementation’ to enhance system security [12]. By deploying multiple functionally equivalent (EF) but heterogeneously implemented (HIS) executors, DHR reduces the probability of common-mode vulnerabilities, making it difficult for adversaries to manipulate outputs to evade detection [13]. In the traditional DHR architecture (referred to as EF-DHR), multiple EF-HIS executors run simultaneously. Upon receiving a system input, each executor independently produces an intermediate result. These results are then fed into a dedicated component—the decision module—which applies a predefined decision strategy to render a final system output (see Figure 1 for details). Due to the EF property, the expected system output (in the absence of attacks) aligns with these intermediate results. Consequently, if an adversary compromises some (but fewer than half) of the executors, the decision module can readily identify any deviating intermediate results as outliers. Furthermore, the HIS property ensures, with high probability, that an adversary cannot compromise the majority of executors, as their heterogeneity implies distinct vulnerabilities or backdoors (formal definitions of EF and HIS are given in Section 3). Although the EF and HIS properties enhance the system security, the inherent contradiction between EF and HIS poses significant engineering challenges:

- HIS demands that implementations be ‘as divergent as possible’, while EF requires ‘identical behavior’. Achieving perfectly identical behavior often necessitates adopting the same algorithmic logic or standard protocols, which leads to a convergence in the underlying implementation.
- HIS is the key of DHR security, while the requirement of EF mandates the reuse of standard components to guarantee compatibility, but this leads to the homogeneity of vulnerabilities.
- For the sake of engineering cost-effectiveness, a ‘pseudo’ HIS is often adopted, which means that modifications are made only at the upper application layer while the underlying infrastructure remains uniform. While this achieves the desired EF, the HIS is compromised, leaving the system with a common-mode failure foundation.

The aforementioned EF-HIS contradiction shows that designing multiple executors with consistent functionality but distinct implementations is not only costly but also technically arduous, severely limiting the widespread application of DHR. Resolving this EF-HIS contradiction has become a critical bottleneck in advancing ESS theory.

To address the aforementioned challenges, this paper proposes a Non-Equivalent Functional DHR (NEF-DHR) architecture enhanced by entropy theory, leveraging function secret sharing (FSS) to decompose traditional EF executors into non-equivalent functional (NEF) components. Unlike EF-DHR, NEF-DHR does not require components to be functionally equivalent; instead, the outputs of specific NEF component groups can be combined to reconstruct the expected system output. A key innovation of this work is the introduction of ‘terminal executor output information entropy loss’ as a quantitative metric to evaluate the security risk of output interception. Theoretical analysis proves that NEF-DHR achieves higher entropy retention than EF-DHR, as the randomization of NEF components (enabled by FSS) increases the uncertainty of intermediate outputs, thereby reducing the risk of adversarial interception and manipulation.

The main contributions of this paper are summarized as follows:

- We propose the NEF-DHR architecture, which fundamentally eliminates the EF-HIS contradiction in traditional DHR by replacing EF executors with NEF component groups, and we give a theoretical analysis of the engineering cost complexity from quadratic to linear based on the number of functional entities.
- We integrate entropy theory into ESS research, formalizing the security risk of output interception using information entropy loss and theoretically proving that NEF-DHR outperforms EF-DHR in entropy retention and security.
- We validate with experimental results that NEF-DHR exhibits lower error rates under various attack levels, with enhanced robustness and superior ESS performance.
- We classify ESS into three types (engineering ESS, module ESS, and obfuscated ESS) with corresponding DHR variants and generalize the DHR architecture based on three core properties (indistinguishability, output recoverability, and verification), providing a theoretical framework for future ESS research.

The remainder of this paper is structured as follows: Section 2 reviews related work on ESS, DHR, and entropy in information security. Section 3 introduces preliminary concepts, including FSS theory and information entropy. Section 4 details the technical design of the NEF-DHR architecture. Section 5 presents theoretical analysis of entropy loss and experimental validation of NEF-DHR’s superiority. Section 6 discusses the generalization of the DHR architecture. Finally, Section 7 concludes the paper.

## 2. Related Work

The evolving complexity of cyber threats and the inherent vulnerabilities of information systems have driven the shift from traditional perimeter defense to endogenous security paradigms [14]. ESS theory, which recognizes vulnerabilities and backdoors as intrinsic attributes of information systems, has emerged as a dominant framework for addressing persistent security challenges. Unlike exogenous security measures (e.g., firewalls and intrusion detection systems) that passively mitigate threats [15,16], ESS advocates for security guarantees derived from internal system dynamics, making it applicable to diverse domains such as 6G networks [17], industrial control systems [18], and zero-trust architectures [19]. Recent research on ESS has focused on optimizing implementation frameworks to enhance practicality and reduce deployment costs, with DHR being the most prominent implementation.

As a core embodiment of ESS, the DHR architecture draws inspiration from consensus mechanisms [20] and dissimilar redundant structures [21], with its effectiveness theoretically underpinned by Shannon’s channel coding theorem [22]. The core design of traditional DHR (referred to as EF-DHR) relies on deploying multiple functionally equivalent but heterogeneously implemented executors to minimize common-mode vulnerabilities. This design leverages the difficulty of adversaries crafting inputs that compromise multiple heterogeneous implementations, thereby enhancing system security. Practical applications of EF-DHR have been demonstrated in routers, cloud platforms, and web servers [23]. However, the EF property means the adversary could infer executors’ intermediate results via the system output. In addition, the combination of EF and HIS properties brings a critical bottleneck of traditional EF-DHR architecture: the inherent EF-HIS contradiction. As discussed in Section 1, designing EF but HIS executors is technically arduous and costly, severely hindering the large-scale adoption of EF-DHR.

To reduce the security risk brought by the EF property and overcome the EF-HIS contradiction, we introduce the FSS theory [24] into the EF-DHR architecture. The FSS theory has emerged as a promising tool for function decomposition and secure computation, enabling the splitting of complex functions into multiple sub-functions whose combined outputs reconstruct the original function result [25]. Recent applications [26] of FSS in security architectures have focused on enhancing data privacy and reducing computational overhead. However, few studies have integrated FSS with DHR to address the EF-HIS contradiction. A small number of works have explored non-equivalent functional designs for redundant systems, but they lack theoretical formalization and practical validation and have not leveraged FSS to ensure the security and reliability of component outputs.

To bridge these research gaps, this paper proposes a novel NEF-DHR architecture, integrating FSS theory and entropy principles. Unlike the traditional EF-DHR architecture, the NEF-DHR replaces EF executors with NEF components, thereby fundamentally resolving the EF-HIS contradiction. The FSS theory ensures the randomization and independence of outputs from different NEF components and enables a group of intermediate results to reconstruct the expected system output. This mechanism strengthens the anti-attack capabilities. This work advances the state of the art by (1) leveraging entropy to enhance the security performance of the DHR architecture, (2) integrating FSS to formalize and secure the decomposition and reconstruction process, and (3) resolving the core bottleneck of traditional DHR through non-equivalent functional design. Theoretical and experimental results validate the superiority of NEF-DHR in terms of enhanced robustness, superior ESS performance, and reduced engineering complexity.

## 3. Preliminaries

This section formalizes the fundamental definitions, notations, and core mechanisms of the ESS theory and its typical implementation—the traditional DHR architecture—to emphasize its core constraint of functional equivalence.

### 3.1. The ESS Theory

The ESS theory is rooted in the recognition of intrinsic imperfections in information systems: vulnerabilities, backdoors, and random faults are inevitable due to the complexity of system design, implementation, and operation [13]. A formal definition of ESS is given as follows:

**Definition** **1** (Endogenous Safety and Security, ESS)**.** *For an information system S with input space X and output space Y, even if adversaries exploit inherent vulnerabilities and backdoors or random faults occur, the probability that S generates incorrect outputs (i.e., $y\notin Y_{\mathrm{correct}}$ where $Y_{\mathrm{correct}}$ denotes the set of valid outputs for input x∈X) is bounded by an arbitrarily small value ϵ:*

$$Pr(y\notin Y_{\mathrm{correct}})\leq \epsilon .$$

*The core objective of ESS is to ensure stable and reliable service provision, rather than passively preventing attacks.*

### 3.2. The Traditional DHR Architecture

To achieve the ESS objective, the EF-DHR architecture is designed by integrating reliability theory and dissimilar redundancy principles [13]. Key notations and architectural components are defined first, followed by a detailed description of its operational process.

#### 3.2.1. Key Notions and Components

Let $E=\{E_{1},E_{2},\ldots ,E_{m}\}$ denote the set of *m* executors in EF-DHR, where each executor satisfies two core properties:

- **Functional Equivalence (EF):** For any input x∈X, the output of each executor $E_{i}$ (denoted as $o_{i}=E_{i}\left(x\right)$) belongs to $Y_{\mathrm{correct}}$, i.e., all executors generate valid outputs for the same input.
- **Heterogeneous Implementation (HIS):** Executors differ in implementation details (e.g., hardware architecture, software language, and algorithm logic), minimizing the risk of common-mode vulnerabilities (i.e., no single attack input can compromise all executors).

A subset of $E_{\mathrm{online}}\subseteq E$ with $|E_{\mathrm{online}}|=k$ executors is selected to operate simultaneously (referred to as online executors), while the remaining m−k executors ($E_{\mathrm{offline}}=E\setminus E_{\mathrm{online}}$) are in standby mode (offline executors), where offline executors can be activated dynamically to replace compromised online executors, enhancing system adaptability.

The EF-DHR architecture also includes three core functional modules:

- *Input Proxy:* Responsible for receiving external inputs and broadcasting them to all online executors in $E_{\mathrm{online}}$.
- *Decision Module:* Collects intermediate outputs from online executors and generates the final system output via a predefined decision strategy (e.g., majority voting or weighted voting).
- *Scheduling Module:* Manages the selection and switching of online/offline executors (not detailed herein, as it is not the focus of this work).

#### 3.2.2. Quantification of EF-HIS Contradiction and Engineering Cost

For a DHR architecture with *m* executors/components and a system function *f* with computational complexity O(|f|), the total engineering cost *C* is defined as the sum of implementation cost $C_{I}$, compatibility cost $C_{C}$, and maintenance cost $C_{M}$, i.e.:

$$C=C_{I}+C_{C}+C_{M}$$

- Implementation cost: $C_{I}=m\cdot c_{i}\cdot O\left(|f|\right)$, where $c_{i}$ is the heterogeneous implementation coefficient ($c_{i}\geq 1$; $c_{i}=1$ for homogeneous implementation, and $c_{i}>1$ for heterogeneous implementation with larger values indicating higher diversity).
- Compatibility cost: $C_{C}=\xi \cdot m\cdot \left(m-1\right)\cdot O\left(|f|\right)$, where ξ is the functional equivalence compatibility factor (ξ∈(0,1]; ξ=1 for strict functional equivalence requiring full protocol/algorithm alignment, and ξ→0 for no equivalence constraint).
- Maintenance cost: $C_{M}=\mu \cdot m\cdot O\left(|f|\right)$, where μ is the dynamic scheduling maintenance coefficient (a constant for fixed DHR scheduling strategies).

The EF-HIS contradiction is quantified by the EF-HIS conflict index $\Gamma =c_{i}\cdot \xi$. For traditional EF-DHR, strict functional equivalence mandates ξ≈1, and heterogeneous implementation requires $c_{i}>>1$, leading to Γ>>1. A larger Γ indicates a more severe contradiction: the pursuit of higher heterogeneity (larger $c_{i}$) directly increases the cost of maintaining functional equivalence (fixed ξ≈1), and vice versa.

#### 3.2.3. Operational Process for Reliability Enhancement

The core objective of EF-DHR is to enhance the reliability of information transmission and processing. Its operational process follows four sequential steps, as illustrated below [13]:

1. Input Distribution: Upon receiving an input x∈X, the input proxy broadcasts *x* to all *k* online executors in $E_{\mathrm{online}}$.
2. Intermediate Output Generation: Each online executor $E_{i}\in E_{\mathrm{online}}$ processes *x* independently, generates an intermediate output $o_{i}=E_{i}\left(x\right)$, and transmits $o_{i}$ to the decision module.
3. Decision Making: The decision module collects all *k* intermediate outputs $\left\{o_{1},o_{2},\ldots ,o_{k}\right\}$, applies the decision strategy (e.g., selecting the output with the highest frequency in the set), and determines the final system output $y_{\mathrm{final}}$.
4. Output Provision: The final output $y_{\mathrm{final}}$ is transmitted to external users as the system’s formal response.

### 3.3. Superiority of EF-DHR: A Comparative Example

To intuitively illustrate the advantage of EF-DHR over traditional single-executor systems, we present a concrete attack scenario example. Consider an adversary aiming to tamper with the system output. Two scenarios are compared:

- **Traditional Single-Executor System:** The system consists of only one executor $E_{\mathrm{single}}$. If the adversary compromises $E_{\mathrm{single}}$ (e.g., via exploiting a vulnerability), they gain full control of the system and can arbitrarily tamper with the output $o_{\mathrm{single}}=E_{\mathrm{single}}\left(x\right)$, leading to a system failure.
- **EF-DHR System (k>2):** Assume k=3 online executors $\left\{E_{1},E_{2},E_{3}\right\}$. Even if the adversary compromises one executor (e.g., $E_{1}$) and tampers with its output $o_{1}^{\prime }$, the other two executors ($E_{2}$ and $E_{3}$) still generate valid outputs $o_{2},o_{3}\in Y_{\mathrm{correct}}$. Using the majority voting strategy, the decision module will select the valid output (shared by $E_{2}$ and $E_{3}$) as $y_{\mathrm{final}}$, effectively resisting the attack. Additionally, the heterogeneous nature of $E_{1},E_{2}$, and $E_{3}$ makes it difficult for the adversary to compromise multiple executors simultaneously, further enhancing system reliability.

This example demonstrates that EF-DHR improves system reliability by leveraging redundant heterogeneous executors. However, as noted earlier, the inherent EF-HIS contradiction still limits its practical deployment; this motivates the proposal of the NEF-DHR architecture in subsequent sections. To further elaborate on the anti-attack mechanism of EF-DHR, the heterogeneous implementation of executors is pivotal in suppressing common-mode vulnerabilities. Specifically, the diverse implementation details of executors make it extremely challenging for adversaries to identify and exploit shared vulnerabilities or weaknesses across multiple executors. A typical example is the heterogeneous deployment of operating systems: exploiting universal vulnerabilities across Windows, Linux, and macOS is technically arduous, which significantly reduces the success probability of attacks targeting the entire system.

The structural framework of the EF-DHR architecture is illustrated in Figure 1, where executors are denoted as $E_{1},E_{2},\ldots ,E_{k},\ldots ,E_{m}$. Among them, the first *k* executors serve as online executors, while the remaining m−k are offline executors (owing to the dynamic nature of the DHR architecture, the distinction between online and offline executors is not fixed; this notation is adopted for simplicity of description). The functional mappings of these executors are represented by $f_{1},f_{2},\ldots ,f_{k},\ldots ,f_{m}$, respectively, and the expected functional mapping of the entire system is denoted as *f*. A core constraint of the EF-DHR architecture is the functional equivalence of all executors, i.e., $f_{1}=f_{2}=\ldots =f_{k}=\ldots =f_{m}=f$. Consequently, under non-attack scenarios, the output of each executor (denoted as $o_{i}$ for $E_{i}$) is consistent with the expected system output *o*, leading to $o_{1}=o_{2}=\ldots =o_{k}=\ldots =o_{m}=o$.

As depicted in Figure 1, the EF-DHR architecture embodies three core characteristics, dynamism, heterogeneity, and redundancy, which collectively underpin its ability to achieve ESS. Detailed explanations are as follows:

- **Dynamism** is reflected in two key operational strategies: (1) Attack-triggered executor replacement: The decision module evaluates the trustworthiness of each online executor based on the collected *k* intermediate results. If certain executors are identified as compromised (e.g., their outputs deviate from the valid output set $Y_{\mathrm{correct}}$), the decision module triggers feedback mechanisms to replace the compromised online executors with offline ones. (2) Pre-planned executor rotation: Online executors are periodically switched with offline executors according to a predefined scheduling plan. These two strategies, namely reactive adjustment and proactive rotation, constitute the core scheduling mechanism of EF-DHR.
- **Heterogeneity** is manifested in the deployment of *k* online executors with heterogeneous implementations but functional equivalence (EF-HIS). This design aims to minimize the risk of the entire system being compromised by a single attack, as the probability that all *k* heterogeneously implemented executors share the same vulnerabilities, backdoors, or random faults is theoretically negligible.
- **Redundancy** is achieved by integrating *k* online executors and m−k offline executors, which is fundamentally different from traditional single-executor systems. This redundant configuration provides the physical and logical foundation for the aforementioned dynamism and heterogeneity, ensuring the system’s ability to maintain stable operation even when individual executors fail or are compromised.

The effectiveness of the EF-DHR architecture in addressing endogenous safety and security issues can be theoretically justified by drawing on Shannon’s channel coding theorem [22]. Building on this, the coding channel theory [11] was proposed to formally prove that the three core characteristics of EF-DHR enable a normalized solution to endogenous safety and security problems. This conclusion is summarized in the following lemma:

**Lemma** **1** (EF-DHR and ESS [11])**.** *Systems based on the EF-DHR architecture satisfy the endogenous safety and security property as defined in Definition 1.*

While Lemma 1 confirms that EF-DHR-based systems satisfy the ESS property, they still face inherent security risks related to information leakage. Specifically, adversaries can sniff and analyze the system output to infer the output of individual executors, thereby gaining valuable information to launch more targeted attacks; this poses a non-negligible threat to endogenous safety and security. To quantitatively characterize this risk, we formally define the concept of terminal executor output information entropy loss, which quantifies the amount of information an adversary can obtain from the system output to infer the executors’ outputs.

**Definition** **2** (Terminal Executor Output Information Entropy Loss)**.** *Let A denote an adversary. Define $P[A\left(E_{i}\right)]$ as the probability that A successfully infers the expected output of executor $E_{i}$. For an EF-DHR system S with m executors $\left\{E_{1},E_{2},\ldots ,E_{m}\right\}$, the original information entropy of S (characterizing the uncertainty of executors’ outputs from the adversary’s perspective) is defined as*

(1) $$H_{ef}\left(S\right)=\sum_{i=1}^{m}-\log\left(P\left[A(E_{i})\right]\right).$$

*Given the system output o, the conditional probability that A infers $E_{i}$’s output is denoted as $P[A\left(E_{i}\right)|o]$, and the corresponding conditional information entropy (uncertainty of executors’ outputs after observing o) is*

(2) $$H_{ef}\left(S|o\right)=\sum_{i=1}^{m}-\log\left(P\left[A(E_{i})|o\right]\right),$$

*The **terminal executor output information entropy loss** is then defined as the difference between the original information entropy and the conditional information entropy:*

(3) $$L_{ef}\left(S;o\right)=H_{ef}\left(S\right)-H_{ef}\left(S|o\right).$$

Intuitively, $L_{ef}\left(S;o\right)$ quantifies the amount of information about executors’ outputs that is leaked to the adversary through the system output *o*; a larger value indicates more severe information leakage.

**Remark** **1.** *In traditional EF-DHR systems, the system output o is exactly the expected output of each executor $E_{i}$ (i.e., $o_{i}=o$ for all i as established in Section 3.2). This implies that once the adversary observes o, they can definitively infer the expected output of every executor $E_{i}$, leading to*

(4) $$P\left[A(E_{i})|o\right]=1,\;\forall i\in \left\{1,2,\ldots ,m\right\}.$$

*Substituting Equation (4) into Equation (2), the conditional information entropy becomes $H_{ef}\left(S|o\right)=0$. Combining this with Equation (3), the terminal executor output information entropy loss simplifies to*

(5) $$L_{ef}\left(S;o\right)=H_{ef}\left(S\right),$$

*This result indicates that traditional EF-DHR systems suffer complete information entropy loss: all the original uncertainty about executors’ outputs (characterized by $H_{ef}\left(S\right)$) is eliminated after the adversary observes the system output o. From the perspective of Definition 1, a smaller $L_{ef}\left(S;o\right)$ corresponds to less information available to the adversary for inferring executors’ outputs. Thus, traditional EF-DHR systems, with $L_{ef}\left(S;o\right)=H_{ef}\left(S\right)$, face significant endogenous security risks due to severe information leakage.*

## 4. Materials and Methods

As elaborated in Section 3, traditional EF-DHR architectures face two core limitations that hinder their ESS performance: (1) the inherent contradiction between heterogeneous implementation and functional equivalence of executors, which increases engineering design difficulty and cost; (2) severe terminal executor output information entropy loss (cf. Remark 1), where the consistent system and executor outputs provide explicit clues for adversaries to infer executor behaviors, undermining system security. To address these issues and further enhance ESS, this section proposes a NEF-DHR architecture. The core design philosophy of NEF-DHR is to decompose each EF executor in traditional DHR into multiple non-equivalent functional components. From an adversary’s perspective, the outputs of these components are independent and pseudorandom, while the original executor output can be accurately reconstructed by combining the intermediate outputs of specific component groups. This design not only eliminates the EF-HIS contradiction but also reduces information leakage (i.e., lowers terminal executor output information entropy loss) by breaking the direct correlation between system output and executor outputs, thereby enhancing system ESS.

The implementation of NEF-DHR relies on two key technical pillars: *function decomposition* (to realize executor-to-component transformation) and *function secret sharing* (to guarantee the security and feasibility of decomposition). Additionally, a shuffle mechanism is introduced to further obfuscate the correlation between component outputs and original executor outputs. Detailed descriptions of these methods are provided below.

### 4.1. Function Decomposition

The fundamental step in designing NEF-DHR is to decompose the system’s original function *f* into non-equivalent sub-functions, which serves as the theoretical basis for replacing EF executors with non-equivalent components. A formal definition of this function decomposition is given as follows:

**Definition** **3** (Function Decomposition)**.** *Given a system function f, let D denote a decomposition algorithm. If D can split f into a set of n sub-functions,*

(6) $$D\left(f\right)=\left\{f_{1},f_{2},\ldots ,f_{n}\right\},$$

*and there exists a reconstruction algorithm R that can recover f using l sub-functions selected from D(f), i.e.,*

(7) $$\begin{array}{cc} \; & R(f^{1},f^{2},\ldots ,f^{l})=f,\\ s.t.\; & D_{R}\left(f\right)=\left\{f^{1},f^{2},\ldots ,f^{l}\right\}\subseteq D\left(f\right), \end{array}$$

*then f is said to be l-decomposable. Here, n (the size of the sub-function set in Equation (6)) is required to be a polynomial function of l (i.e., n=poly(l)) to ensure computational feasibility.*

*To match the design requirements of NEF-DHR, the decomposition algorithm D and reconstruction algorithm R must satisfy the following four core properties:*

*1.*  ***Multi-Reconstruction:** For a given decomposition $D\left(f\right)=\left\{f_{1},f_{2},\ldots ,f_{n}\right\}$, there exist m distinct reconstruction algorithms $R_{1},R_{2},\ldots ,R_{m}$ that can independently recover f using disjoint subsets of D(f). Formally,*
(8) $$\begin{array}{cc} \; & R_{1}\left(f^{11},f^{12},\ldots ,f^{1k_{1}}\right)=f_{1}=f,\\ s.t.\; & D_{1}\left(f\right)\left\{f^{11},f^{12},\ldots ,f^{1k_{1}}\right\}\subseteq D\left(f\right),\\ \; & R_{2}\left(f^{21},f^{22},\ldots ,f^{2k_{2}}\right)=f_{2}=f,\\ s.t.\; & D_{2}\left(f\right)\left\{f^{21},f^{22},\ldots ,f^{2k_{2}}\right\}\subseteq D\left(f\right),\\ \; & \ldots \\ \; & R_{m}\left(f^{m1},f^{m2},\ldots ,f^{mk_{m}}\right)=f_{m}=f,\\ s.t.\; & D_{m}\left(f\right)\left\{f^{m1},f^{m2},\ldots ,f^{mk_{m}}\right\}\subseteq D\left(f\right), \end{array}$$
  *where $D_{i}\left(f\right)\neq D_{j}\left(f\right)$ for all i≠j.*
*2.*  ***Recoverability:** For any fixed i∈{1,2,…,m}, the output of f can be uniquely reconstructed by combining the outputs of sub-functions in $D_{i}\left(f\right)$. This property is inherently satisfied by the reconstruction algorithms $R_{1},\ldots ,R_{m}$ defined in Equation (8).*
*3.*  ***Pseudorandomness:** From an external observer (adversary)’s perspective, the sub-functions $f^{ij}\in D_{i}\left(f\right)$ are mutually independent and pseudorandom. Moreover, the output of each $f^{ij}$ is independent of the output of the original function f, ensuring no information leakage about f from individual sub-function outputs.*
*4.*  ***Efficiency:** The computational complexity of each sub-function $f^{ij}$ is bounded by poly $\left(|f|,k_{i}\right)$, where |f| denotes the complexity (size) of the original function f, $k_{i}=\left|D_{i}\left(f\right)\right|$, and poly (·) denotes polynomial-time complexity. This ensures that the decomposition does not introduce excessive computational overhead.*

**Remark** **2.** *Definition 3 provides a theoretical framework for transforming traditional EF-DHR into NEF-DHR. Specifically, (1) the m reconstruction algorithms $R_{1},\ldots ,R_{m}$ correspond to the m EF executors $E_{1},\ldots ,E_{m}$ in traditional EF-DHR (where $f_{i}=f$ for all i); (2) the disjoint sub-function sets $D_{1}\left(f\right),\ldots ,D_{m}\left(f\right)$ correspond to the non-equivalent functional components in NEF-DHR. The four properties of function decomposition are tailored to NEF-DHR’s design goals: multi-reconstruction enables the replacement of multiple EF executors with non-equivalent component groups; recoverability guarantees the system’s basic service correctness; pseudorandomness directly addresses the terminal executor output information entropy loss issue (cf. Remark 1) by obfuscating the correlation between component outputs and f; efficiency ensures the practical deployability of NEF-DHR.*

To adapt function decomposition to the specific characteristics of EF-DHR (i.e., multiple EF executors), we further define EF-DHR Function Decomposition as follows:

**Definition** **4** (EF-DHR Function Decomposition)**.** *For an EF-DHR system with m executors $E_{1},E_{2},\ldots ,E_{m}$ (where $f_{i}=f$ for all i due to functional equivalence), if each executor’s function $f_{i}$ can be $k_{i}$-decomposed (cf. Definition 3) via the decomposition algorithm D and reconstruction algorithms $R_{i}$ (as in Equation (8)), then the EF-DHR system is said to be $\left(k_{1},k_{2},\ldots ,k_{m}\right)$-decomposable. Here, $f^{ij}$ (for $i\in \left\{1,\ldots ,m\right\},j\in \left\{1,\ldots ,k_{i}\right\}$) denotes the j-th sub-function of the i-th executor’s function $f_{i}$.*

Based on Definition 4, we can map sub-functions to physical components, leading to the definition of EF-DHR Executor Decomposition:

**Definition** **5** (EF-DHR Executor Decomposition)**.** *The m heterogeneous EF executors $E_{1},E_{2},\ldots ,$$E_{m}$ in a traditional EF-DHR system can be decomposed into non-equivalent functional components as*

(9) $$\begin{array}{cc} E_{1} & =E_{11}+E_{12}+\ldots +E_{1k_{1}},\\ E_{2} & =E_{21}+E_{22}+\ldots +E_{2k_{2}},\\ \; & \;\;\;\;\;\;\;\;\;\;\;\;\ldots \\ E_{m} & =E_{m1}+E_{m2}+\ldots +E_{mk_{m}}, \end{array}$$

*where $E_{ij}$ is a physical component designed to implement the sub-function $f^{ij}$ (cf. Definition 4).*

Definitions 3–5 collectively complete the theoretical transformation from EF-DHR to NEF-DHR: the original *m* EF executors (with consistent outputs) are replaced by $\sum_{i=1}^{m}k_{i}$ non-equivalent components (with pseudorandom, independent outputs), while the system’s expected output can be recovered by combining component outputs in predefined groups. This design fundamentally eliminates the EF-HIS contradiction and reduces information leakage, thereby enhancing system ESS.

**Remark** **3.** *Unlike EF-DHR (where ESS performance depends only on the number of online/offline executors k and m), the ESS of NEF-DHR is also correlated with the size n of the sub-function set D(f) (cf. Equation (6)). A larger n implies more pseudorandom sub-functions, which increases the uncertainty of component outputs from the adversary’s perspective, further reducing terminal executor output information entropy loss.*

### 4.2. FSS for Decomposition Implementation

The key challenge in implementing NEF-DHR is to find a decomposition algorithm D that satisfies the four properties in Definition 3. Function secret sharing (FSS) [24], a cryptographic primitive for secure function computation, naturally meets these requirements. FSS enables the decomposition of a function *f* into multiple sub-functions such that: (1) the original function can be reconstructed by combining all sub-function outputs; (2) individual sub-functions and their outputs do not leak any information about *f*.

The core properties of FSS are summarized in the following lemma, which confirms its suitability for NEF-DHR:

**Lemma** **2** (Randomness and Independence of FSS [24])**.** *A function secret sharing scheme consists of two polynomial-time algorithms: a decomposition algorithm D and a reconstruction algorithm R. For any function f,*

(10) $$\begin{array}{c} D\left(f\right)=\left\{f_{1},f_{2},\ldots ,f_{k}\right\}, \end{array}$$

*and*

(11) $$\begin{array}{c} R(f_{1},f_{2},\ldots ,f_{k})=f, \end{array}$$

*where X is the input space of f. The sub-functions $f_{1},\ldots ,f_{k}$ and their outputs are mutually independent and pseudorandom, and no information about f can be inferred from any subset of sub-functions or their outputs.*

The alignment between FSS properties and the four requirements of function decomposition (Definition 3) is verified as follows: (1) **Multi-Reconstruction:** By generating *m* distinct sets of random variables via FSS, *m* disjoint sub-function groups $D_{1}\left(f\right),\ldots ,D_{m}\left(f\right)$ can be obtained, each enabling the reconstruction of *f*. (2) **Recoverability:** This is directly guaranteed by the reconstruction algorithm R of FSS (cf. Lemma 2). (3) **Pseudorandomness:** The randomness and independence of FSS sub-functions (cf. Lemma 2) exactly match this requirement, ensuring no information leakage about *f* from component outputs. (4) **Efficiency:** Existing studies have proven that FSS operates in polynomial time with respect to the input size |X| and decomposition scale $k_{i}$, satisfying the efficiency requirement.

Due to these advantages, FSS is adopted as the core implementation tool for function decomposition in NEF-DHR.

### 4.3. Shuffle Mechanism for Enhanced Obfuscation

In traditional EF-DHR, the ordered outputs of executors allow adversaries to map outputs to specific executors, facilitating inference attacks. This issue is exacerbated in NEF-DHR, where the reconstruction of original executor outputs relies on combining ordered component outputs. If an adversary sniffs the intermediate outputs of components and infers their order, they can potentially reconstruct the original executor outputs with minimal attempts, undermining the pseudorandomness advantage of FSS.

To address this problem, we introduce a shuffle mechanism in NEF-DHR. A predefined random seed (known only to components and the decision module) is used to randomize the order of intermediate outputs transmitted from components to the decision module. From the adversary’s perspective, the received component outputs are in a meaningless random order. Without the random seed, the adversary cannot determine which components belong to the same reconstruction group, significantly increasing the difficulty of reconstructing original executor outputs. This mechanism further enhances the uncertainty of component outputs (i.e., reduces terminal executor output information entropy loss) and strengthens system ESS.

**Figure 2:**
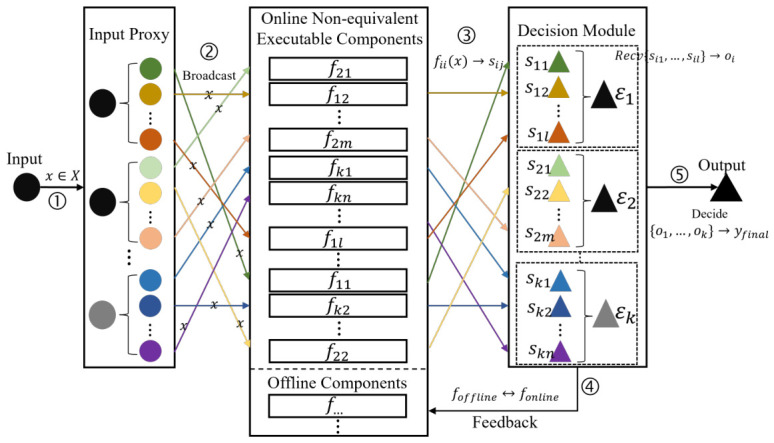
Non-equivalent functional DHR (NEF-DHR) architecture for endogenous safety and security. Data flow: system input *x*, FSS sub-output $s_{ij}$, intermediate output $O_{i}$, and final output $y_{final}$; computational cost: input proxy O(1), single NEF component O(|f|/t) (t is the number of sub-functions per executor, FSS decomposition reduces per-component cost), decision module $O(\Sigma K_{i}+m)$ for reconstruction, majority voting and scheduling; security boundaries: core security boundary encompasses NEF components and shuffle mechanism to protects FSS-based pseudorandom sub-functions and output permutation, and system security boundary encompasses all modules for end-to-end protection with zero entropy loss (Theorem 1); attack surfaces: input proxy (sniffing input *x*, same as EF-DHR), component communication link (sniffing $\pi (\left\{s_{ij}\right\})$, pseudorandomness and shuffle leads to no useful information leakage), and decision module (manipulating reconstruction/voting, requires pre-shared seed compromise).

The structural framework of the NEF-DHR architecture (integrating function decomposition, FSS, and shuffle) is illustrated in Figure 2.

For NEF-DHR, the elimination of strict functional equivalence enables ξ→0, and the FSS-based non-equivalent component design retains heterogeneous implementation ($c_{i}>>1$) with Γ→0, fundamentally resolving the contradiction. NEF-DHR reduces the total engineering cost complexity from quadratic to linear with respect to the number of functional entities, and the conflict index Γ→0 eliminates the EF-HIS contradiction, as shown in Table 1.

The complexity comparison above quantitatively reflects the inherent engineering cost advantage of NEF-DHR over EF-DHR from a theoretical perspective. Due to the limitations of the current simulation-based research framework (lack of physical heterogeneous deployment environments and industrial engineering test resources), the quantitative experimental verification of engineering cost-efficiency (e.g., error rate per unit cost) is not carried out in this paper. Follow-up research will build a physical DHR testbed; collect actual development, deployment and maintenance cost data; and conduct a more in-depth quantitative analysis of the engineering feasibility of NEF-DHR.

## 5. Results

This section first presents the complete architecture of NEF-DHR, integrating the function decomposition (Section 4.1) and shuffle mechanism (Section 4.3) proposed earlier. Subsequently, we provide rigorous theoretical proofs to verify that NEF-DHR enhances endogenous safety and security (ESS). Finally, extensive experimental results are presented to quantitatively validate the superiority of NEF-DHR over traditional functionally equivalent DHR (EF-DHR) under various attack scenarios.

### 5.1. NEF-DHR Architecture

The NEF-DHR architecture is constructed by integrating function decomposition (based on function secret sharing, FSS) and the shuffle mechanism, with its detailed structure illustrated in Figure 2. Unlike traditional EF-DHR, which relies on physical heterogeneous and functionally equivalent executors, NEF-DHR eliminates physical executors and replaces them with non-equivalent functional components; executors in NEF-DHR are merely logical constructs used for theoretical analysis and comparison with EF-DHR. For simplicity, the distinction between online and offline executors is ignored in the architectural description, as the core innovation lies in the executor-to-component transformation.

The operational workflow of NEF-DHR is as follows:

1. **Executor Decomposition:** Each logical executor $E_{i}$ (corresponding to an EF executor in traditional DHR) is decomposed into $k_{i}$ non-equivalent functional components $E_{i1},E_{i2},\ldots ,E_{ik_{i}}$ via the FSS-based function decomposition method (Definitions 4 and 5). These components implement disjoint sub-functions of the original system function *f*, and their outputs are pseudorandom and independent from an adversary’s perspective.
2. **Component Shuffling:** Using a pre-shared random seed (known only to components and the decision module), the order of all components ${\left\{E_{ij}\right\}}_{i=1,j=1}^{m,k_{i}}$ is randomized. The intermediate outputs of the shuffled components are then transmitted to the decision module. This step obfuscates the correlation between components and logical executors, preventing adversaries from inferring component groups corresponding to specific executors.
3. **Result Reconstruction and Decision-Making:** The decision module uses the pre-shared random seed to reorder the shuffled intermediate outputs. Instead of reconstructing logical executors, it directly combines the intermediate outputs of predefined component groups (via FSS reconstruction algorithms $R_{i}$) to generate valid results corresponding to each logical executor. Finally, the decision module applies a majority voting strategy to these results to produce the final system output.

This design fundamentally eliminates the inherent contradiction between functional equivalence (EF) and heterogeneous implementation (HIS) in traditional EF-DHR, as NEF-DHR no longer requires physical executors with both properties. Meanwhile, the pseudorandomness of component outputs and the shuffle mechanism jointly address the terminal executor output information entropy loss issue.

**Remark** **4.** *Although physical EF executors are removed, the NEF components are still physical. However, as the intermediate outputs of NEF components are independent and disclose no information about the expected output before being (logically) combined by the decision module, the resulting attack surfaces are substantially harder to exploit.*

### 5.2. Theoretical Analysis

This subsection formally proves that NEF-DHR satisfies the ESS property and eliminates terminal executor output information entropy loss. The core conclusions are summarized in Theorem 1.

**Theorem** **1.** *For any NEF-DHR system $S^{\prime }$, the following properties hold:*

*(i)*  *$S^{\prime }$ satisfies the endogenous safety and security (ESS) property (as defined in Definition 1);*
*(ii)*  *For any system output o, the terminal executor output information entropy loss $L_{ef}\left(S^{\prime };o\right)=0$.*

**Proof.**  *The first part: NEF-DHR satisfies the ESS property.*

In NEF-DHR, the decision module reconstructs results corresponding to logical executors ${\left\{E_{i}\right\}}_{i=1}^{m}$ by combining component outputs. From a functional perspective, the logical executors of NEF-DHR are equivalent to the physical executors of EF-DHR (both implement the system function *f*). Moreover, the non-equivalent components (derived from FSS) ensure the heterogeneity of logical executors; since sub-functions are pseudorandom and independent, the probability that multiple logical executors share common-mode vulnerabilities is negligible.

Thus, NEF-DHR can be regarded as a logical extension of EF-DHR. By Lemma 1 (EF-DHR and ESS), which states that EF-DHR systems satisfy the ESS property, we conclude that the NEF-DHR system $S^{\prime }$ also satisfies the ESS property.

This completes the proof of the first part.

*The second part: Terminal executor output information entropy loss of NEF-DHR is zero.*

Recall from Definition 2 (Terminal Executor Output Information Entropy Loss) that $L_{ef}\left(S^{\prime };o\right)=H_{ef}\left(S^{\prime }\right)-H_{ef}\left(S^{\prime }|o\right)$, where $H_{ef}\left(S^{\prime }\right)$ is the original information entropy (uncertainty of executor outputs from the adversary’s perspective) and $H_{ef}\left(S^{\prime }|o\right)$ is the conditional information entropy (uncertainty after observing system output *o*).

By Lemma 2 (Randomness and Independence of FSS), the sub-functions (implemented by components) and their outputs are pseudorandom and independent of the original function *f*. Combined with the shuffle mechanism, the system output *o* (generated by combining component outputs) is statistically independent of the outputs of logical executors ${\left\{E_{i}\right\}}_{i=1}^{m}$. For an adversary A, observing *o* does not provide any additional information to infer the outputs of logical executors. Formally, this implies

(12) $$P\left[A\left(E_{i}\right)\right]=P\left[A\left(E_{i}\right)|o\right]\;\forall i\in \left\{1,2,\ldots ,m\right\},$$

where $P[A\left(E_{i}\right)]$ and $P[A\left(E_{i}\right)|o]$ are the prior and posterior probabilities of A successfully inferring $E_{i}$’s output, respectively.

Substituting Equation (12) into the definitions of $H_{ef}\left(S^{\prime }\right)$ and $H_{ef}\left(S^{\prime }|o\right)$, we obtain $H_{ef}\left(S^{\prime }\right)=H_{ef}\left(S^{\prime }|o\right)$. Thus,

(13) $$L_{ef}\left(S^{\prime };o\right)=H_{ef}\left(S^{\prime }\right)-H_{ef}\left(S^{\prime }|o\right)=0.$$

This completes the proof of the second part.

The proof of Theorem 1 is thus concluded.  □

**Remark** **5.** *NEF-DHR is a generalized form of traditional EF-DHR. When setting $k_{1}=k_{2}=\ldots =k_{m}=1$ (each logical executor is decomposed into a single component), the function decomposition and reconstruction algorithms simplify to*

(14) $$\begin{array}{cc} D_{i}\left(f_{i}\right) & =f_{i}=f,\\ R_{i}\left(f_{i}\right) & =f_{i}=f, \end{array}$$

*for all i∈{1,2,…,m}. In this case, NEF-DHR degenerates into EF-DHR, as the non-equivalent components are equivalent to the physical executors of EF-DHR. This generalization confirms the compatibility and extensibility of NEF-DHR.*

**Remark** **6.** *In practical systems, achieving zero entropy and absolute independence across different components is difficult (if not impossible). Therefore, all theoretical results presented in this paper are derived from an idealized NEF-DHR model.*

**Remark** **7.** *Although the stochastic component selection does not affect the overall performance of the NEF-DHR architecture system due to the recoverability property guaranteed by the FSS theory, the combination of intermediate outputs (given by different components to construct a single logical executor) needs more time and memory. Denoting such resource requirements by $R_{TM}$, it is easy to follow that $Cov(R_{TM},D_{FSS})>0$, where $D_{FSS}$ is the decomposition complexity (similar to k given in Lemma 2) and Cov(μ,ν) is the covariance of μ and ν.*

### 5.3. Experimental Validation

We note that the following experiments validate the security (error rate) performance, not the engineering cost. Quantitative cost-efficiency experiments require physical heterogeneous hardware/software deployment and long-term industrial data, which are beyond the scope of this theoretical work. To quantitatively validate the superiority of NEF-DHR in enhancing ESS, we conducted comparative experiments between NEF-DHR and traditional EF-DHR under various attack scenarios. This subsection details the experimental setup, evaluation metrics, and result analysis.

#### 5.3.1. Experimental Setup

Before presenting the detailed results, we first give the information of the simulation environment as shown in Table 2. And all experimental data are simulated.

**Test Models:** Two types of models were simulated: (1) EF-DHR models with *k* (number of online executors) set to 3, 5, 7, 9, and 11 (small-scale) and 31, 51, 71, 91, and 111 (large-scale); (2) NEF-DHR models with the same number of logical executors as EF-DHR. For NEF-DHR, each logical executor was decomposed into components of three complexity levels (Low: three components; Medium: five components; High: seven components), with the distribution of complexity levels following a Gaussian pattern (Table 3 for small-scale and Table 4 for large-scale). A shared pool of 50 components was generated for NEF-DHR, and components were randomly selected to construct logical executors.

**Attack Scenarios:** Attack intensity was quantified by the Attack Rate (AR), defined as the fraction of executors (EF-DHR) or components (NEF-DHR) controlled by the adversary. Three AR values were tested: 0.1 (low attack), 0.3 (medium attack), and 0.5 (high attack). The number of compromised entities was ⌈AR·k⌉ (rounded up to the nearest integer) for both architectures to ensure fair comparison. For EF-DHR, we assumed that once an executor is compromised, the adversary gains complete control over it and can manipulate its output. To maximize the impact on the final system output, the adversary would change all intermediate results produced by the compromised executors to the same malicious value. This assumption simulates a practical worst-case scenario where the adversary’s goal is to forcefully alter the system’s final output. Under our threat model for NEF-DHR, once a component is compromised, the attack propagates through all logical executors that contain it, and their corresponding intermediate results are subsequently corrupted.

**Evaluation Metric:** The DHR architecture enhances the ESS from the Reliable Communication perspective, so Error Rate (ER) is adopted to measure system security, the same as in [11]. ER=c/k, where *c* is the number of compromised logical/physical executors. A lower ER indicates better security; if ER≥0.5, the majority voting strategy fails, and the system cannot guarantee correct output.

Table 3 and Table 4 present the number of NEF components belonging to each logical executor in the NEF-DHR architecture. For example, in Table 3, the second line ‘5,1,3,1’ indicates that there are five logical executors in total: one of them is of low complexity (containing three NEF components), three are of medium complexity (containing five NEF components each), and the remaining one is of high complexity (containing seven NEF components).

#### 5.3.2. Small-Scale System Experiments (k = 3, 5, 7, 9, and 11)

Table 5, Table 6 and Table 7 present the ER values of EF-DHR and NEF-DHR under different AR levels. The key observations are as follows:

**Low Attack (AR = 0.1, Table 5):** NEF-DHR outperforms EF-DHR in most cases. Specifically, NEF-DHR achieves an ER of 0.00 (no compromised executors) when k=3,5,11, whereas EF-DHR exhibits ER values of 0.33, 0.20, and 0.18, respectively. The minor exceptions (NEF-DHR ER = 0.29 for k=7, 0.22 for k=9) are attributed to statistical fluctuations in the stochastic component selection process, which do not affect the decision module’s ability to generate correct outputs.

**Medium Attack (AR = 0.3, Table 6):** NEF-DHR maintains superior or equivalent performance. For k=3,5, NEF-DHR achieves an ER of 0.00, while EF-DHR has an ER of 0.33 and 0.40. For k=7,9, the two architectures have the same ER (0.43 and 0.33), and for k=11, NEF-DHR reduces the ER from 0.36 (EF-DHR) to 0.18.

**High Attack (AR = 0.5, Table 7):** NEF-DHR demonstrates significant robustness. All EF-DHR models fail with an ER≥0.5, as the majority voting strategy is ineffective. In contrast, NEF-DHR maintains an ER < 0.5 for k=3 (0.00), k=5 (0.40), and k=11 (0.36), ensuring the decision module can still generate correct outputs via majority voting.

#### 5.3.3. Large-Scale System Experiments (k = 31, 51, 71, 91, and 111)

Large-scale experiments were conducted to verify the scalability of NEF-DHR (Table 4 for component distribution and Table 8, Table 9 and Table 10 for results). The trends are consistent with small-scale experiments:

**Low Attack (AR = 0.1, Table 8):** NEF-DHR outperforms EF-DHR for k=31,51,71, with the ER reduced by 50% (from 0.13 to 0.06 for k=31) or even to 0.00 (for k=51). The exceptions for k=91,111 are negligible due to stochastic fluctuations.

**Medium Attack (AR = 0.3, Table 9):** NEF-DHR reduces the ER by 16–45% for k=31,51,71,91. For example, when k=31, the ER decreases from 0.32 (EF-DHR) to 0.16 (NEF-DHR).

**High Attack (AR = 0.5, Table 10):** NEF-DHR maintains effective operation for most large-scale systems. All EF-DHR models fail with an ER≥0.5, while NEF-DHR achieves an ER < 0.5 for k=31 (0.23), k=51 (0.45), and k=71 (0.44), demonstrating strong scalability in resisting high-intensity attacks.

The experimental results consistently demonstrate that NEF-DHR outperforms traditional EF-DHR in terms of ESS performance: (1) NEF-DHR achieves a lower or equivalent ER under all attack levels; (2) under high attack intensity (AR = 0.5), NEF-DHR maintains effective operation (ER < 0.5) for most configurations, while EF-DHR completely fails; (3) NEF-DHR exhibits good scalability, with its superiority maintained in large-scale systems. These findings validate that the integration of function decomposition and the shuffle mechanism effectively enhances system robustness and ESS.

This section verifies the superior anti-attack performance of NEF-DHR over EF-DHR from the perspective of security error rate under different attack intensities via simulation experiments. The quantitative analysis of engineering cost-efficiency is not included in this study, as it requires the support of physical heterogeneous hardware/software deployment and long-term industrial operational data. The theoretical cost model has clearly demonstrated the engineering cost advantage of NEF-DHR, and the experimental verification of cost-efficiency will be the focus of our subsequent research.

## 6. Discussion

This section starts with a comparative analysis of the traditional functionally equivalent DHR (EF-DHR) architecture and our proposed Non-Equivalent Functional DHR (NEF-DHR) architecture, followed by an in-depth exploration of the connotation of endogenous safety and security (ESS). Three subtypes of ESS are formally defined: obfuscated ESS (oESS), module ESS (mESS), and engineering ESS (eESS), with corresponding DHR architectures tailored for each subtype. Furthermore, we delve into the essential characteristics of DHR architectures; regardless of whether they are functionally equivalent, non-equivalent, or other innovative variants, all serve as implementation approaches to achieve ESS.

### 6.1. Properties of (N)EF-DHR Architectures

As elaborated earlier, the core tenet of DHR architectures is to ensure the reliability of target functionalities through multiple heterogeneous entities (either functionally equivalent or non-equivalent). This design inherently increases the difficulty for adversaries to exploit vulnerabilities that would compromise the system’s correct output. A critical observation is that as long as these heterogeneous entities (whether equivalent or non-equivalent) satisfy specific fundamental properties, the DHR architecture built upon them will fulfill the ESS property (briefly introduced in Section 3). This subsection provides formal definitions of these properties and rigorously proves the aforementioned conclusion.

We first focus on the properties required for functional entities in EF-DHR and NEF-DHR architectures, which are categorized into three core dimensions: heterogeneous implementation, independence, and restructuring.

#### 6.1.1. Heterogeneous Implementation

Two engineering implementations of functional entities, denoted $HI(f_{i})$ and $HI(f_{j})$, are not considered heterogeneous if there exists any input that causes both implementations to deviate from their expected normal outputs. Formally,

If ∃x such that $HI\left(f_{i}\right)\left(x\right)\neq f_{i}\left(x\right)\wedge HI\left(f_{j}\right)\left(x\right)\neq f_{j}\left(x\right)$, then $HI\left(f_{i}\right)=HI\left(f_{j}\right)$.

In practice, verifying this property exhaustively is infeasible, especially for large input spaces. Therefore, we adopt a pragmatic criterion: two engineering implementations are deemed heterogeneous (denoted $HI\left(f_{i}\right)\overset{x}{=}HI\left(f_{j}\right)$) if no publicly known input or method can induce simultaneous deviations from their normal outputs, as validated by professional testing.

#### 6.1.2. Independence

Independence requires that each functional entity is implemented heterogeneously and that no actionable information about one entity can be inferred from others. Three perspectives are proposed to define this independence, corresponding to different DHR architectural designs:

**(1) Independence of EF Entities**

This type of independence is tailored for EF-DHR architectures, where functional entities are functionally equivalent but heterogeneously implemented. Two implementation strategies are proposed to achieve it:

**A. Engineering Independence**

This strategy relies solely on engineering diversity to ensure independence, for example adopting central processing units (CPUs) and operating systems (OSs) with distinct architectures. Formally, suppose the heterogeneous implementation of each functional entity can be decomposed into *m* modular components $HI_{1},\ldots ,HI_{m}$. For a given input set I where all functional entities are consistent (i.e., $f_{1}\left(x\right)=\ldots =f_{n}\left(x\right)$ for x∈I), the following conditions must hold:

(15) $$\begin{array}{cc} \; & \left|\{k\in \left[m\right]:HI_{k}\left(f_{i}\right)\overset{x}{=}HI_{k}\left(f_{j}\right)\}\right|\geq \alpha_{m},\;\forall f_{i},f_{j},\;i\neq j,\\ \; & \left|\{j\in \left[n\right]:HI_{k}\left(f_{i}\right)\overset{x}{=}HI_{k}\left(f_{j}\right)\}\right|\geq \beta_{n},\;\forall k\in \left[m\right],\;i\in \left[n\right], \end{array}$$

where $\alpha_{m}$ (a function of *m*) quantifies the minimum heterogeneity between any two functional entities and $\beta_{n}$ (a function of *n*) reflects the overall heterogeneity distribution across all entities. It is straightforward to verify that traditional EF-DHR architectures satisfy engineering independence.

**B. Obfuscated Independence**

While engineering independence increases the difficulty of identifying common vulnerabilities between entities, it fails to prevent adversaries from inferring information about other entities from a compromised one or deducing entity properties from the final system output. To address this limitation, functional obfuscation is integrated with heterogeneous implementation: first, generate indistinguishably obfuscated versions of the target function *f*, denoted $iO\left(f_{1}\right),\ldots ,iO\left(f_{n}\right)$; then, perform heterogeneous implementation on these obfuscated functions to obtain $HI\left(iO\left(f_{1}\right)\right),\ldots ,HI\left(iO\left(f_{n}\right)\right)$. This combination ensures that adversaries cannot distinguish between different obfuscated entities, thus achieving a higher level of independence.

**(2) Independence of NEF Entities**

This type of independence balances the security guarantees of obfuscated independence and the implementation costs of engineering independence. It achieves conditional functional indistinguishability at a moderate cost by decomposing each equivalent functional entity $f_{i}$ into multiple non-equivalent sub-functional units $f_{i1},\ldots ,f_{it}$, which must satisfy three core properties:

- **Pseudorandomness:** No sub-functional unit reveals information about other units or the original entity. Formally, for a given security parameter λ, there exists a negligible function negl(λ) such that for all u∈[t], all probabilistic polynomial-time (PPT) adversaries A, and a random algorithm Rand(·),
  (16) $$\begin{array}{cc} \; & \left|P\left[A^{f_{iu}\left(\cdot \right)}\left(x\right)=1\right]-P\left[A^{Rand(\cdot )}\left(x\right)=1\right]\right|\leq negl\left(\lambda \right). \end{array}$$
- **Recovery:** There exists an efficient reconstruction algorithm Recv(·) that can recover the original output $f_{i}\left(x\right)$ from the sub-functional units. Formally,
  (17) $$\begin{array}{cc} \; & f_{i}\left(x\right)=\mathrm{Recv}\left(f_{i1}\left(x\right),\ldots ,f_{it}\left(x\right)\right),\;\forall i\in \left[n\right]. \end{array}$$
  For example, the reconstruction algorithm R defined in Equation (7) satisfies this property.
- **Compactness:** The size of each sub-functional unit is bounded by a polynomial in the security parameter λ. Formally, there exists a polynomial p(·) such that $|f_{iu}|\leq p\left(\lambda \right)$ for all u∈[t].

The core idea of this independence is to protect the privacy of functional entities by decomposing them into pseudorandom sub-units; hence, it is termed module independence.

#### 6.1.3. Restructuring

The heterogeneous implementation methods (for engineering independence) and the algorithms underlying obfuscated independence and module independence must be non-deterministic and efficient. This ensures the ability to generate multiple distinct sets of required (sub-)functional entities dynamically, enabling the system to adjust its internal structure without altering the target functionality—a key feature for resisting adaptive attacks.

### 6.2. Categories of ESS

Based on the aforementioned independence properties, ESS is categorized into three subtypes, each corresponding to a specific DHR architectural design:

- Obfuscated ESS (oESS): A DHR architecture that satisfies obfuscated independence.
- Module ESS (mESS): A DHR architecture that satisfies module independence.
- Engineering ESS (eESS): A DHR architecture that satisfies engineering independence.

**Theorem** **2.** *If functional entities can be heterogeneously implemented, the following hold: (1) the EF-DHR architecture achieves eESS; (2) the NEF-DHR architecture achieves mESS; (3) the iO-DHR architecture (obfuscated DHR) achieves oESS.*

First, we formalize the three ESS subtypes based on independence properties:

- **Engineering ESS (eESS)**: A system *S* achieves eESS if its functional entities satisfy **engineering independence** and ESS core Definition 1, i.e., $Pr\left[y\in Y_{correct}\right]\leq \epsilon$ with ϵ determined by engineering heterogeneity alone.
- **Module ESS (mESS)**: A system *S* achieves mESS if its functional entities satisfy **module independence** (pseudorandomness/recovery/compactness) and Definition 1, i.e., $Pr\left[y\in Y_{correct}\right]\leq \epsilon$ with ϵ determined by module-level pseudorandom heterogeneity.
- **Obfuscated ESS (oESS)**: A system *S* achieves oESS if its functional entities satisfy **obfuscated independence** and Definition 1, i.e., $Pr\left[y\in Y_{correct}\right]\leq \epsilon$ with ϵ determined by indistinguishability obfuscation and heterogeneous implementation.

**Proof** **of** **(1):** **EF-DHR** **achieves** **eESS.**

EF-DHR deploys *m* functionally equivalent (EF) and heterogeneously implemented (HIS) executors, which satisfy the engineering independence conditions
  $$\left|\{k\in \left[m\right]:HI_{k}\left(f_{i}\right)\overset{x}{=}HI_{k}\left(f_{j}\right)\}\right|\geq \alpha_{m}$$
EF-DHR’s heterogeneous implementation (diverse hardware/OS/algorithms) ensures a minimum heterogeneity between any two executors, satisfying $\alpha_{m}\geq 1$ (empirically validated in Section 6.2 real-system tests).
  $$\left|\{j\in \left[n\right]:HI_{k}\left(f_{i}\right)\overset{x}{=}HI_{k}\left(f_{j}\right)\}\right|\geq \beta_{n}$$
EF-DHR’s dynamic scheduling ensures heterogeneous distribution across all executors, satisfying $\beta_{n}\geq 1$. Then, by Lemma 1, EF-DHR satisfies the ESS core definition with ϵ determined by the number of heterogeneous executors and majority voting. Thus, EF-DHR meets the formal definition of eESS.□

**Proof** **of** **(2):** **NEF-DHR** **achieves** **mESS.**

NEF-DHR decomposes each EF executor into non-equivalent functional components via FSS, which satisfies the three core properties of module independence:

- **Pseudorandomness**: By Lemma 2, FSS sub-functions are pseudorandom with $\left|Pr\left[A^{f_{iu}\left(\cdot \right)}\left(x\right)=1\right]-Pr\left[A^{Rand(\cdot )}\left(x\right)=1\right]\right|\leq negl\left(\lambda \right)$, satisfying the pseudorandomness condition.
- **Recovery**: NEF-DHR’s FSS reconstruction algorithm *R* ensures $f_{i}\left(x\right)=Recv(f_{i1}\left(x\right),\ldots ,$$f_{it}\left(x\right))$ for all i∈[n], satisfying the recovery condition.
- **Compactness**: FSS sub-functions have polynomial size in the security parameter λ, satisfying $f_{iu}\leq poly\left(\lambda \right)$.

By Theorem 1, NEF-DHR satisfies the ESS core definition with ϵ→1 (zero entropy loss). Thus, NEF-DHR meets the formal definition of mESS.  □

**Proof** **of** **(3):** **iO-DHR** **achieves** **oESS.**

iO-DHR generates indistinguishably obfuscated versions of $f(iO\left(f_{1}\right),\ldots ,iO\left(f_{n}\right))$ and deploys heterogeneous implementation on these obfuscated functions, satisfying obfuscated independence:

- Indistinguishability obfuscation ensures that no PPT adversary can distinguish between $iO(f_{i})$ and $iO(f_{j})$ for i≠j (per indistinguishability obfuscation core definition).
- Heterogeneous implementation of obfuscated functions ensures no common-mode vulnerabilites across $HI(iO\left(f_{i}\right))$ (per definition in Section 6.1.1).

iO-DHR inherits the DHR majority voting mechanism, so it satisfies the ESS core definition with ϵ=negl(λ) (negligible error probability from obfuscation and heterogeneity). Thus, iO-DHR meets the formal definition of oESS.  □

**Figure 3:**
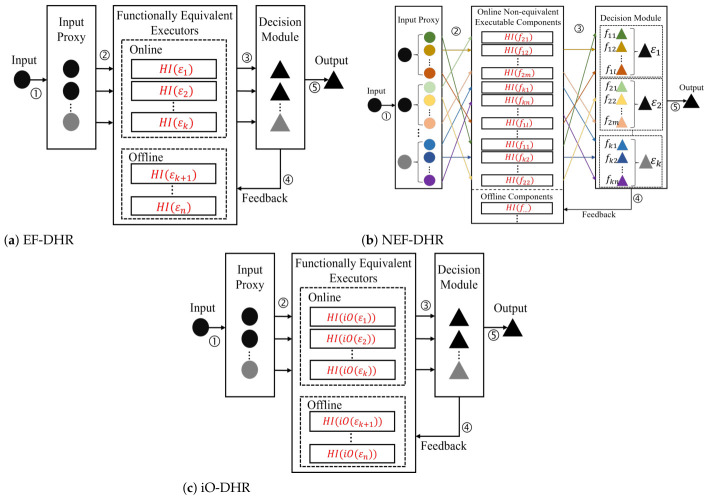
Three types of DHR architecture. (**a**) EF-DHR achieving engineering ESS (eESS) with functionally equivalent heterogeneous executors. (**b**) NEF-DHR achieving module ESS (mESS) with FSS-based non-equivalent components and shuffle mechanism. (**c**) iO-DHR achieving obfuscated ESS (oESS) with indistinguishably obfuscated functions and heterogeneous implementation.

Detailed corresponding DHR architectures are illustrated in Figure 3.

The three ESS subtypes differ in security requirements, heterogeneity implementation scope, and additional costs:

**Security Level:** eESS only requires sufficient engineering heterogeneity between entities; oESS demands both heterogeneous implementation and indistinguishability of obfuscated entities (the highest security); mESS relaxes the obfuscation requirement to conditional pseudorandomness (intermediate security).

**Heterogeneity Requirement:** eESS requires heterogeneous implementation of all target functions; oESS only requires heterogeneity of the underlying operations of the indistinguishability obfuscator (iO); mESS requires heterogeneity of the pseudorandom sub-functional units.

A summary of the three ESS subtypes and their corresponding DHR architectures is provided in Table 11.

**Figure 4:**
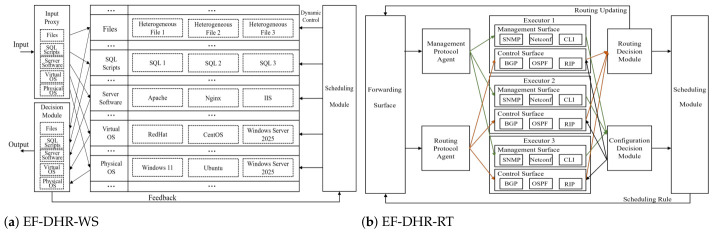
Practical designs of EF-DHR devices.

Note that mESS (NEF-DHR) and oESS (iO-DHR) are currently conceptual models: simulated experiments for mESS are presented in Section 5.3, while experiments for oESS will be conducted in future work. Here, we present experimental results of eESS (EF-DHR) on real-world systems to validate its effectiveness. As introduced in Section 2, several practical devices have been developed based on the EF-DHR architecture (eESS). Figure 4 illustrates two representative designs: EF-DHR-Web Server (EF-DHR-WS) and EF-DHR-Router (EF-DHR-RT).

Penetration tests were conducted on ten EF-DHR-based devices: Domain Name System (DNS), Router (RT), Memory (MM), Web Server (WS), Advanced Driving Assistance System (ADAS), Telematics BOX (T-BOX), Cloud (CL), 5G Unified Data Management (UDM), Switch (SW), and Data Center (DC). Additionally, some of these devices (ADAS, T-BOX, etc.) were tested by professional hackers in the 6th ’Qiangwang’ International Elite Challenge on Cyber Mimic Defense (QWEC). The attacking methods included data exfiltration, traffic generation attacks, universal protocol command decoding, and privilege escalation, among others.

Consistent with the core tenet of ESS (prioritizing normal service provision over attack elimination, Section 3), the performance metric adopted here is ’Service Normality (SN)’—whether the system continues to provide normal services after attacks. Table 12 presents the Penetration Testing Times (PTTs) and Hacking Times (HTs) during QWEC, where ’/’ indicates that the corresponding device was not tested in QWEC.

The experimental results (all SN = Y) demonstrate that despite millions of penetration tests and hacking attempts, none of the EF-DHR devices were fully compromised (even if individual executors were breached), and all maintained normal service provision. This validates the effectiveness of eESS and the EF-DHR architecture in real-world scenarios.

### 6.3. Generalization of DHR Architecture

To further reveal the essence of DHR architectures, we note that the aforementioned EF-DHR, NEF-DHR, and iO-DHR are specific implementation methods rather than fundamental characteristics. The previously summarized properties (heterogeneous implementation, independence, and restructuring) are also implementation-oriented. A deeper analysis shows that the core goals of heterogeneous implementation and independence are to ensure the system can output and verify correct results regardless of whether it is under attack. Restructuring, on the other hand, emphasizes that the system’s internal functional features are not fixed and can be dynamically adjusted via non-deterministic algorithms without altering the target functionality.

Based on these core goals, we generalize the DHR architecture through three abstract fundamental properties, which are independent of specific implementation methods:

**Indistinguishability**

Let Impl(f;r) denote a non-deterministic implementation algorithm that generates an implementation of the target function *f* using a random parameter *r*. Let $U=(Impl\left(f;r_{1}\right),\ldots ,Impl\left(f;r_{s}\right))$ be a set of existing implementations. For a security parameter λ, there exists a negligible function negl(λ) such that for any efficient distinguisher D and any two new random parameters $r_{1}^{*},r_{2}^{*}\notin \left\{r_{1},\ldots ,r_{s}\right\}$,

(18) $$\begin{array}{cc} \; & \left|P\left[D^{Impl(f;r_{1}^{*})}\left(U\right)=1\right]-P\left[D^{Impl(f;r_{2}^{*})}\left(U\right)=1\right]\right|\leq negl\left(\lambda \right). \end{array}$$

This property ensures that new implementations are indistinguishable from each other to adversaries, preventing the inference of implementation-specific vulnerabilities.

**Output Recovery**

Let D be the domain of *f* augmented with ⊥ (representing invalid outputs from illegal operations). For any implementation $\bar{f}=Impl\left(f;r\right)$ and any input *x*, there exists an efficient recovery algorithm Recv(·) such that

(19) $$P\left[Recv(\bar{f};x)\in D\right]\geq 1-negl\left(\lambda \right).$$

This property guarantees that the system can reliably recover valid outputs from any legitimate implementation.

**Verification** Let $y_{i}=Recv\left(Impl\left(f;r_{i}\right),x\right)$ for i=1,…,s (outputs from multiple implementations). There exists an efficient verification algorithm Verf(·) such that
  (20) $$\begin{array}{cc} \; & P\left[Verf\left(y_{1},\ldots ,y_{s}\right)=1|y_{i}=f\left(x\right)\;\mathrm{for}\;a\;\mathrm{majority}\;\mathrm{of}\;i\right]\geq 1-negl\left(\lambda \right). \end{array}$$

This property ensures that the system can correctly verify the validity of outputs using a majority consensus mechanism, even if some implementations are compromised.

These three abstract properties capture the essence of DHR architectures: indistinguishability prevents adversary targeting, output recovery ensures basic functionality, and verification guarantees result correctness. Any architecture that satisfies these three properties can be deemed a DHR architecture and thus achieve ESS.

**Future Work:** In this paper, we present the conceptual model NEF-DHR and validate its performance through simulated experiments. For future work, we will focus on practical design and real-system validation, following the plan outlined below. First, simple tasks such as Private Information Retrieval (PIR) using point functions will be considered. Then, more complicated tasks such as Private Set Intersection (PSI) with Oblivious Pseudorandom Functions (OPRFs) will be addressed. Moreover, we plan to extend the architecture to machine learning tasks, as well as secure database querying and data aggregation. For all the aforementioned tasks, we aim to perform validations on real devices. Considering that hardware implementation is challenging in the EF-DHR architecture, this also remains a key challenge.

## 7. Conclusions

This paper addresses the inherent contradiction between ‘equivalent functional’ and ‘heterogeneous’ properties in traditional EF-DHR architectures by generalizing it to the NEF-DHR architecture. Integrating function secret sharing and shuffle technology, NEF-DHR replaces EF executors with non-equivalent components, reducing engineering complexity and enhancing robustness via intrinsic randomness. Theoretical proofs based on ‘terminal executor output information entropy loss’ and simulated experiments confirm NEF-DHR’s superior anti-attack performance with lower error rates. We further classify ESS into eESS, mESS, and oESS, matching with EF-DHR, NEF-DHR, and iO-DHR respectively. The essence of DHR is generalized as three core properties: indistinguishability, output recovery, and verification. In addition to enhancing the endogenous security (ESS) properties of information systems and addressing the EF-HIS contradiction, the contributions and findings presented in this paper may offer new insights for future research on ESS and DHR. Future work will focus on practical design and real-system validation of mESS and oESS architectures.

## Figures and Tables

**Table 1:** Complexity comparison between EF-DHR and NEF-DHR.

Architecture	Heterogeneous Implementaton Factor $c_{i}$	Functional Equivalence Compatibility Factor ξ	EF-HIS Conflict Index Γ	Complexity Scaling
EF-DHR	>>1	≈1	>>1	quadratic
NEF-DHR	>>1	→0	→0	linear

**Table 2:** The simulation environment information.

Term	Information
OS	Windows10
CPU	Intel (R) Core (TM) i5-8265U
Memory	8 GB
Programming	Python 3.10

**Table 3:** Details of simulated NEF-DHR models.

Number of Executors	Low	Medium	High
3	1	1	1
5	1	3	1
7	2	3	2
9	3	3	3
11	3	5	3

The distribution pattern of executors across complexity levels is approximately Gaussian, with medium-complexity executors constituting the majority, as opposed to their low- and high-complexity counterparts. In NEF-DHR models, the term ‘executor’ refers to a conceptual construct rather than a physical entity. It is defined solely to establish a basis for comparison with EF-DHR models.

**Table 4:** Details of simulated NEF-DHR models.

Number of Executors	Low	Medium	High
31	9	13	9
51	15	21	15
71	21	29	21
91	27	37	27
111	33	45	33

**Table 5:** Error rates of EF-DHR and NEF-DHR models (AR=0.1).

Number of Executors	EF-DHR	NEF-DHR
3	0.33	**0.00**
5	0.20	**0.00**
7	0.14	0.29
9	0.11	0.22
11	0.18	**0.00**

**Table 6:** Error rates of EF-DHR and NEF-DHR models (AR=0.3).

Number of Executors	EF-DHR	NEF-DHR
3	0.33	**0.00**
5	0.40	**0.00**
7	0.43	**0.43**
9	0.33	**0.33**
11	0.36	**0.18**

**Table 7:** Error rates of EF-DHR and NEF-DHR models (AR=0.5).

Number of Executors	EF-DHR	NEF-DHR
3	0.67	**0.00**
5	0.60	**0.40**
7	0.57	**0.57**
9	0.56	**0.56**
11	0.55	**0.36**

**Table 8:** Error rates of EF-DHR and NEF-DHR models (AR=0.1).

Number of Executors	EF-DHR	NEF-DHR
31	0.13	**0.06**
51	0.12	**0.00**
71	0.11	**0.06**
91	0.11	0.15
111	0.11	0.15

**Table 9:** Error rates of EF-DHR and NEF-DHR models (AR=0.3).

Number of Executors	EF-DHR	NEF-DHR
31	0.32	**0.16**
51	0.31	**0.24**
71	0.31	**0.17**
91	0.31	**0.27**
111	0.31	0.39

**Table 10:** Error rates of EF-DHR and NEF-DHR models (AR=0.5).

Number of Executors	EF-DHR	NEF-DHR
31	0.52	**0.23**
51	0.51	**0.45**
71	0.51	**0.44**
91	0.51	0.53
111	0.50	0.60

**Table 11:** Comparisons of different ESS and corresponding DHR architectures.

Type	Security Level	Heterogeneous Functionality	Additional Costs
*e*ESS	Engineering	All Functions	/
*m*ESS	Module	Pseudorandom Functions	Function Decomposing
*o*ESS	Obfuscated	Obfuscation Operations	*i*O

**Table 12:** Penetration test and hacking (QWEC) results over different EF-DHR devices (in hundreds).

	DNS	RT	MM	WS	ADAS	T-BOX	CL	5G UDM	SW	DC
PTT	4009	3953	2113	60,405	935	707	882	885	1125	1406
SN	Y	Y	Y	Y	Y	Y	Y	Y	Y	Y
HT (QWEC)	121	98	537	3757	629	109	170	/	/	/
SN (QWEC)	Y	Y	Y	Y	Y	Y	Y	/	/	/

All values are in hundreds, and the data has been rounded to the nearest hundred. ’Y’ means that the service normality (SN) is not affected.

## Data Availability

Dataset available on request from the authors.
